# Tissue plasminogen activator modified thromboelastography identifies fibrinolysis resistance in dogs with immune-mediated hemolytic anemia

**DOI:** 10.3389/fvets.2025.1571683

**Published:** 2025-06-05

**Authors:** Robert Goggs, Samantha Davis, Marjory B. Brooks

**Affiliations:** ^1^Department of Clinical Sciences, Cornell University College of Veterinary Medicine, Ithaca, NY, United States; ^2^Comparative Coagulation Laboratory, Department of Population Medicine and Diagnostic Sciences, Cornell University College of Veterinary Medicine, Ithaca, NY, United States; ^3^BluePearl Pet Hospital, Atlanta, GA, United States

**Keywords:** immunothrombosis, neutrophil extracellular traps, nucleosomes, cell-free DNA, thrombin-activatable fibrinolysis inhibitor, plasminogen activator inhibitor-1

## Abstract

**Introduction:**

Immune-mediated hemolytic anemia (IMHA) is an important immunologic disorder in dogs that is associated with high mortality rates, frequently due to thromboembolism. Multiple factors contribute to the pathophysiology of thrombosis in IMHA including intravascular tissue factor expression, platelet activation, and neutrophil extracellular trap (NET) formation. It was hypothesized that dogs with IMHA have impaired fibrinolysis that can be detected using a modified viscoelastic assay and that biomarkers of NET formation are associated with this hypofibrinolysis.

**Methods:**

Twenty dogs with non-associative IMHA were enrolled and paired thromboelastography (TEG) assays with and without additional tissue plasminogen activator (tPA) performed. A panel of hemostasis tests including measurement of plasma thrombin-activatable fibrinolysis inhibitor (TAFI) activity, active plasminogen activator inhibitor-1 (PAI-1), and concentrations of cell-free DNA (cfDNA) and nucleosomes were also performed.

**Results:**

Dogs with IMHA had hypercoagulable TEG tracings, increased TAFI activity and frequently displayed fibrinolysis resistance defined as minimal lysis in tPA augmented TEG assays. Increased concentrations of cfDNA, nucleosomes and active PAI-1 in dogs with IMHA compared to healthy controls were identified.

**Discussion:**

These observations support the hypothesis that hypofibrinolysis is a common feature of IMHA in dogs. Increased plasma active PAI-1 concentrations and TAFI activities might contribute to the observed hypofibrinolysis. The combined hypercoagulability and hypofibrinolysis observed supports recent recommendations to provide thromboprophylaxis to all dogs with IMHA. These findings also suggest that NETosis might contribute to the common prothrombotic imbalance of IMHA in dogs.

## Introduction

Immune-mediated hemolytic anemia (IMHA) is an important immunologic disorder in dogs (1) that is associated with high mortality rates (2–9). Thrombosis and thromboembolism are major causes of death in dogs with IMHA (4), often manifest as macrothrombi in the splenic and portal veins (10, 11) and as pulmonary thromboembolism (12, 13). The inciting cause of thrombus formation in IMHA is multifactorial, including procoagulant effects of hemolysis (14) and immunothrombosis involving inflammation-induced intravascular tissue factor (TF) expression, platelet activation, and the generation of procoagulant microparticles (14–16). The underlying mechanisms of thrombotic disease and risk of thrombosis for individual dogs with IMHA remain ill-defined and hinder optimization of effective antithrombotic drug regimens (17). Whole blood viscoelastic tests of coagulation are considered global assessments of hemostasis that incorporate the contribution of erythrocytes, platelets, plasma proteins and microparticles (18). The viscoelastic method thromboelastography (TEG) has identified features of hypercoagulability in several studies of dogs with IMHA (19–21). As routinely performed, TEG assays are insensitive measures of fibrinolysis and are unable to evaluate thrombotic risk due to hypofibrinolysis. Modified TEG assays, configured with the addition of a tissue plasminogen activator (tPA) reagent, have been developed to better characterize impaired fibrinolysis (22).

Autoantibody-mediated hemolysis is proinflammatory (23), and dogs with IMHA typically have marked neutrophilia (8). Extracellular DNA decorated with citrullinated histones and proteins, including myeloperoxidase and neutrophil elastase, released from activated neutrophils are referred to as extracellular traps (NETs) (24, 25), in a process termed NETosis (26). Concentrations of circulating cell-free DNA (cfDNA) and nucleosomes have been studied as surrogate markers for NETs (27–32), and NETosis has been observed in dogs with IMHA (33–35). Thrombin generation and clot formation might be potentiated by NETs, which can also impair fibrinolysis, thereby increasing the risk of pathologic immunothrombosis (36, 37). Nucleosomes facilitate thrombus formation in platelet-dependent and platelet-independent manners (38), and impair clot dissolution by inhibiting fibrinolysis (39). Intravascular cfDNA is procoagulant because of its anionic surface charge (40). It also inhibits fibrinolysis by forming a ternary complex with fibrin and plasmin (41), and by accelerating inactivation of tPA by plasminogen-activator inhibitor-1 (PAI-1) (29). These factors combine to increase the risk of pathologic thrombosis. As such, NETs are therapeutic targets in IMHA (34, 42). Since one mechanism of heparin therapy is to scavenge histones (43), this might contribute to the beneficial effect of individually dose-adjusted heparin in dogs with IMHA (44).

Although they have been studied separately, coagulation, fibrinolysis and NETosis have not been simultaneously evaluated in dogs with IMHA, a knowledge-gap the present study sought to fill. It was hypothesized that dogs with IMHA have fibrinolysis-resistant clots as identified by tPA modified TEG and that biomarkers of NETosis are associated with this hypofibrinolysis.

## Materials and methods

### Sample size calculations

Plasma cfDNA concentration data from a study of dogs with IMHA (35) were used to estimate required sample size (G*Power 3, Heinrich-Heine-Universität Düsseldorf) (45). In that study, non-survivors had mean cfDNA concentrations of 1,345 ± 355 ng/mL compared to 1,020 ± 180 ng/mL in survivors. Using the average standard deviation, sample size calculations suggested 20 dogs would be required to detect an equivalent difference with 80% power at *p* < 0.05.

### Animals

This was a single-center prospective observational cohort study conducted from 03/2019 to 05/2023. Dogs fulfilling criteria supportive or diagnostic of non-associative IMHA admitted to the institution hospital were eligible for enrollment. The diagnosis of IMHA was per the 2019 ACVIM guidelines (46), and required anemia (PCV <30%) with spherocytosis or in-saline agglutination or a positive direct antiglobulin test and one of the following: hemoglobinemia, hemoglobinuria, hyperbilirubinemia, or circulating erythrocyte ghosts. Dogs were excluded if they weighed <5 kg, had evidence of an underlying predisposing condition such as tick-borne disease or cancer, or had received glucocorticoids for ≥72 h prior to enrollment. Dogs were also ineligible if they had received heparin, a blood product transfusion, or immunosuppressive therapy other than glucocorticoids within a month of presentation. Dogs were enrolled with written, informed client consent under an approved IACUC protocol (Cornell University 2014-0053). Patient data including age, sex, breed, bodyweight, mental status, capillary refill time, mucous membrane color, temperature, heart rate, respiratory rate, non-invasive blood pressure and the presence or absence of fluid in body cavities, were recorded to enable calculation of the acute patient physiologic and laboratory evaluation (APPLE_full_) (47), and the canine hemolytic anemia objective score (CHAOS) (8). Results of thoracic and abdominal imaging and data on urinalysis performed as part of usual clinical care were extracted from the medical record to confirm that no underlying predisposing conditions existed. Therapeutic management after sample collection was not standardized and attending clinicians determined patient management, including blood product administration. Outcome was recorded as survival to hospital discharge and survival to 28 days post-enrollment to coincide with the peak risk period for mortality in IMHA (5, 6, 8).

### Routine clinicopathologic testing

Blood samples were collected upon initial intravenous catheter placement whenever possible, or by direct venipuncture when necessary. Point-of-care blood lactate concentrations were analyzed immediately in heparinized whole blood (RapidPoint 500, Siemens Healthcare, Malvern, PA). In the following order, blood was then collected directly into evacuated tubes (Vacutainer, BD and Co., Franklin Lakes, NJ) containing no-additive for serum biochemistry, 3.2% sodium citrate (1:9 ratio) for biomarker measurements and coagulation testing including thromboelastography (TEG), and K_2_-EDTA for complete blood counts (CBC). Point-of-care testing for tick-borne diseases (SNAP 4Dx Plus, IDEXX) was performed at presentation using heparinized whole blood. As soon as possible and always within 72 h of sample collection, automated CBCs (ADVIA 2120, Siemens Healthcare) with blood smear review by board-certified veterinary clinical pathologists and serum biochemistry profiles (Cobas C501, Roche Diagnostics, Indianapolis, IN) were analyzed at an American Association of Veterinary Laboratory Diagnosticians accredited laboratory (Animal Health Diagnostic Center, Cornell University). Citrate samples not required for TEG assays were centrifuged for 10 min at 1,370 g (Ultra-8V Centrifuge, LW Scientific, Lawrenceville, GA). To minimize freeze–thaw cycling during subsequent analysis, citrate plasma samples were aliquoted into 1.5 mL freezer tubes (Polypropylene Screw-Cap Microcentrifuge Tubes, VWR, Radnor, PA) with particular care taken to avoid disruption of the cell pellet and frozen immediately at −80°C until batch analysis.

### Rotational viscoelastic coagulation assays

Thromboelastography (TEG) assays were performed with two TEG 5000 analyzers (Haemonetics, Braintree, MA) using kaolin (Haemonetics) and recombinant human TF (Dade Innovin, Siemens Healthineers, Malvern, PA; 1:3,600 final dilution) as activators according to the PROVETS guidelines (48). Modified TEG assays were performed as previously described by addition of tPA (Alteplase, Genentech, South San Francisco, CA, 50 U/mL final in-cup concentration) (49). Assays were conducted in pairs on individual analyzers, for example kaolin + vehicle control in channel 1, kaolin + tPA in channel 2. Detailed TEG assay protocols are available as Supplementary Data Sheet S1. From the TEG tracings the following parameters were recorded: split point (SP), reaction time (R-time), clot formation time (K-time), clot formation angle (alpha angle), maximum amplitude (MA), the G-value (where G = (5,000 × MA)/(100 – MA)), clot lysis at 30 and 60 min (CL30, LY30, CL60 and LY60). For each agonist, the paired tracings with and without tPA were then compared to derive delta lysis values as follows: ΔCL30 (%) = CL30 no tPA − CL30 with tPA; ΔCL60 (%) = CL60 no tPA − CL60 with tPA; ΔLY30 (%) = LY30 with tPA − LY30 no tPA; ΔLY60 (%) = LY60 with tPA − LY60 no tPA. The occurrence of fibrinolysis resistance was defined in three ways for analysis. (i) Using a previously established definition where fibrinolysis resistance was considered present if LY30 values in the tPA-potentiated tracings were smaller than the lowest values established for healthy controls (22, 50). (ii) Comparisons between paired tracings with and without additional tPA with <5% difference in LY30 values defining fibrinolysis resistance. (iii) Comparisons between paired tracings with and without additional tPA with <1% difference in LY30 values defining fibrinolysis resistance.

### Plasma coagulation assays

Analyses of routine coagulation screening tests and hemostatic proteins in citrate plasma were performed at an AAVLD accredited reference laboratory (Comparative Coagulation Laboratory, AHDC) as previously described (51). Determinations of activated partial thromboplastin time (aPTT; Actin FS, Siemens Healthineers), prothrombin time (PT; Thromboplastin LI, Helena Diagnostics, Beaumont, TX) and clottable fibrinogen (STA Fibrinogen, Diagnostica Stago, Parsippany, NJ) were measured using an automated analyzer (STA Compact Max, Diagnostica Stago) equipped with a mechanical endpoint detection system. Fibrinogen concentrations were reported as mg/dL. Antithrombin (AT) activity was measured using a chromogenic substrate kit (Stachrom AT III, Diagnostica Stago) calibrated using a dog plasma standard that was assigned an AT value of 100%. Plasma D-dimer concentrations were measured using a quantitative, turbidimetric immunoassay (HemosIL D dimer, Werfen, Bedford, MA) with human D-dimer calibration standards and controls (HemosIL D-dimer calibrator, Werfen). Concentrations of D-dimer were reported as ng/mL (DDU).

Plasma concentrations of active PAI-1 were determined using a canine-specific ELISA kit (Molecular Innovations, Novi, MI). The assay is configured such that functionally active plasma PAl-1 binds to a urokinase-coated microtiter plate. Latent or complexed PAl-1 does not bind urokinase and is washed away prior to addition of an anti-PAl-1 antibody. After a subsequent wash step, bound primary antibody is detected using a secondary antibody conjugated to horseradish peroxidase. A colorimetric substrate is then added such that the color generated is directly proportional to the concentration of active PAl-1 in the sample. The requisite (but not supplied) buffers were made per the kit manufacturer’s specifications using standard laboratory reagents (Sigma-Aldrich, St. Louis, MO; ThermoFisher Scientific, Waltham, MA). Assays were conducted according to the kit manufacturer’s instructions with all 30-min incubation steps occurring at room temperature with orbital shaking (ThermoMixer FP, Eppendorf). Plate washing was performed using an automated plate washer (50TS Microplate Washer, BioTek Agilent). Light absorbance at 450 nm was measured using a microplate reader (Cytation 1, BioTek Agilent) and unknown concentrations derived from curves plotted using manufacturer-supplied canine PAI-1 standards (Gen5, BioTek Agilent). Quantitation of thrombin activatable fibrinolysis inhibitor (TAFI) activity was performed in duplicate in 96-well microtiter plates using a kinetic chromogenic assay kit (Pefakit TAFI, dsm-firmenich, Parsippany, NJ) as previously described (52), and the mean value of the two replicates used for analysis. This assay employs a thrombin-thrombomodulin complex reagent to activate TAFI present in the test plasma, which acts upon a synthetic chromogenic substrate. Light absorbance at 405 nm was monitored every 10 s for 5 min in an automated plate reader (Cytation 1, BioTek Agilent), activity plotted against the maximum rate of change in light absorbance and TAFI activity expressed as a percentage of a pooled human plasma calibrator. Where measured TAFI activity was above the standard curve, the corresponding activity of the highest available calibrator was used for analysis and the data considered to be non-parametric. Plasma active PAI-1 concentration and TAFI activity in dogs with IMHA were compared with those of 18 healthy beagle dogs sampled for generation of a pooled normal canine plasma standard under a separate IACUC protocol (#2007-0146).

### NETosis biomarkers

Plasma concentrations of nucleosomes and cfDNA were measured as markers of NETosis. Nucleosome concentrations scaled against pooled normal canine plasma were measured using a commercial assay kit (Cell Death Detection ELISA Plus, Roche Diagnostics, Indianapolis, IN) and equipment (Cytation 1, BioTek Santa Clara, CA) as previously described (53). Pooled normal canine plasma was used to scale the nucleosome concentrations because the ELISA lacks a reference standard. Concentrations are expressed as fold over control. Concentrations of cfDNA were measured using a benchtop fluorimeter and relevant reagents (Invitrogen, Qubit 3.0 and dsDNA HS Assay Kit, both ThermoFisher Scientific, Waltham, MA) according to the manufacturers’ instructions as previously described (54).

### Data analysis

Prior to test selection, data were assessed for normality and descriptive statistics calculated as appropriate. Parametric data are presented as mean ± standard deviation, non-parametric data are presented as median (interquartile range). Ordinal data such as the APPLE_full_ and CHAOS scores were assumed to be non-parametric. The paired TEG variables with and without tPA were compared using Wilcoxon signed-rank tests corrected for multiple comparisons using the Holm–Šídák method. Continuous variables were compared between groups using *t*-tests or the Mann–Whitney *U* test as appropriate. Correlations between coagulation parameters were assessed using Spearman’s coefficients and scatterplots. Strength of correlation was defined as follows: <0.5 weak, 0.5–0.6 mild, 0.6–0.7 moderate, 0.7–0.8 strong, 0.8–0.9 very strong, and 0.9–1.0 excellent. Alpha was set at 0.05. Statistical analyses were conducted using commercial software (Prism 10.4.1, GraphPad, Boston, MA).

## Results

### Animals

The study enrolled 20 dogs with primary (non-associative) IMHA, consisting of 14 spayed female dogs and six castrated male dogs of 15 different pure breeds (Boxer *n* = 2, Shih Tzu *n* = 2, Australian Cattle Dog, Border Collie, Boston Terrier, Coonhound, Golden Retriever, German Shepherd Dog, Havanese, Husky, Labrador Retriever, Pit Bull Terrier, Pomeranian, Pug and Standard Poodle all *n* = 1) and three dogs of mixed breed. Patient demographics are summarized in Table 1. Dogs received a median of 2 (1–3) packed red blood cell transfusions of a mean 28 ± 14 mL/kg. The median APPLE_full_ score was 22 (20–25), corresponding to an expected mortality rate of 4% per the original derivation study (47). Complete blood count and serum biochemistry panel data were consistent with previously reported populations of dogs with IMHA (Tables 2, 3). Of the 20 dogs enrolled, 17 (85%) survived to hospital discharge and 16 (80%) were alive 28 days post-enrollment. The small number of deaths precluded meaningful comparison of clinicopathologic data and coagulation variables between survivors and non-survivors.

**Table 1:** Summary demographics and initial physiologic assessments.

Variable	Patient values
Age (y)	8.2 ± 3.3
Bodyweight (kg)	22.8 ± 11.9
T (°F)	103 ± 1.7
HR (bpm)	137 ± 23
RR (bpm)	30 (28–40)
MGCS (3–18)	18 (18–18)
Lactate (mmol/L)	2.7 (2.2–3.6)
Glucose (mg/dL)	103 (88–123)
SAP (mmHg)	153 ± 25
DAP (mmHg)	85 ± 22
MAP (mmHg)	111 ± 17
PCV (%)	20 ± 6
APPLE_full_ (Max. 80)	22 (20–25)
CHAOS (Max. 7)	4 (3–4)

APPLE, acute patient physiologic and laboratory evaluation; CHAOS, canine hemolytic anemia objective score; DAP diastolic arterial pressure; HR, heart rate; MAP, mean arterial pressure; MGCS, modified Glasgow coma scale; PCV, packed cell volume; RR, respiratory rate; SAP systolic arterial pressure; T, temperature.

**Table 2:** Summary complete blood count data.

Variable	Patient values	Reference interval
Hematocrit (%)	19.5 ± 5.7	41–58
Hemoglobin concentration (g/dL)	6.5 ± 1.7	14.1–20.1
Red blood cells (×10^6^/μL)	2.6 ± 0.7	5.7–8.5
Mean corpuscular volume (fL)	74.2 ± 4.4	64–76
Mean corpuscular hemoglobin (pg)	23.9 ± 2.0	21–26
Mean corpuscular hemoglobin concentration (g/dL)	32.2 ± 2.6	33–36
Red-cell distribution width (%)	17.2 ± 4.1	10.6–14.3
Reticulocyte percentage (%)	8.5 (6.7–12.0)	0.2–1.5
Absolute reticulocyte count (×10^3^/μL)	224 (166–331)	11–92
Nucleated red blood cells (/100 WBC)	9 (5–21)	0–1
Total leukocytes (×10^3^/μL)	22.6 ± 13.1	5.7–14.2
Neutrophils (×10^3^/μL)	16.5 ± 8.9	2.7–9.4
Band neutrophils (×10^3^/μL)	1.1 (0.4–2.2)	0.0–0.1
Lymphocytes (×10^3^/μL)	1.5 (1.1–1.9)	0.9–4.7
Monocytes (×10^3^/μL)	1.3 (0.6–2.9)	0.1–1.3
Eosinophils (×10^3^/μL)	0.14 ± 0.19	0.1–2.1
Basophils (×10^3^/μL)	0 (0–0)	0.0–0.1
Platelets (×10^3^/μL)	201 (118–301)	186–545
Mean platelet volume (fL)	27.2 ± 9.4	8.4–14.1

**Table 3:** Summary serum biochemistry panel data.

Variable	Patient values	Reference interval	Variable	Patient values	Reference interval
Sodium	145 ± 5	143–150	ALT	50 (32–180)	17–95
Potassium	3.7 ± 0.6	4.1–5.4	AST	88 (33–224)	18–56
Chloride	110 ± 6	106–114	ALP	178 (118–385)	7–115
Bicarbonate	13.4 ± 3.8	14–24	GGT	0 (0–4)	0–8
AG	24 (22–26)	17–27	Total bilirubin	1.4 (0.7–2.2)	0.0–0.2
Urea nitrogen	21 ± 11	9–26	Direct bilirubin	0.3 (0.2–0.6)	0.0–0.1
Creatinine	0.6 ± 0.2	0.6–1.4	Indirect bilirubin	1.2 (0.5–1.9)	0.0–0.1
Calcium	9.5 ± 0.5	9.4–11.1	Amylase	1,200 (677–1,327)	322–1,310
Phosphate	3.8 ± 0.8	2.7–5.4	Lipase	160 (49–582)	15–228
Magnesium	1.7 (1.6–1.8)	1.5–2.1	Cholesterol	299 ± 57	136–392
Total protein	6.6 ± 0.7	5.5–7.2	CK	262 (120–554)	64–314
Albumin	3.3 ± 0.4	3.2–4.1	LDH	386 ± 278	24–388
Globulin	3.3 ± 0.7	1.9–3.7	Iron	122 (78–180)	97–263
Glucose	92 (76–116)	68–104	TIBC	484 ± 190	280–489

AG, anion gap; ALP, alkaline phosphatase; ALT, alanine transaminase; AST, aspartate transaminase; CK, creatine kinase; GGT, gamma-glutamyl transferase; LDH, lactate dehydrogenase; TIBC, total iron binding capacity.

### Thromboelastography

**Figure 1:**
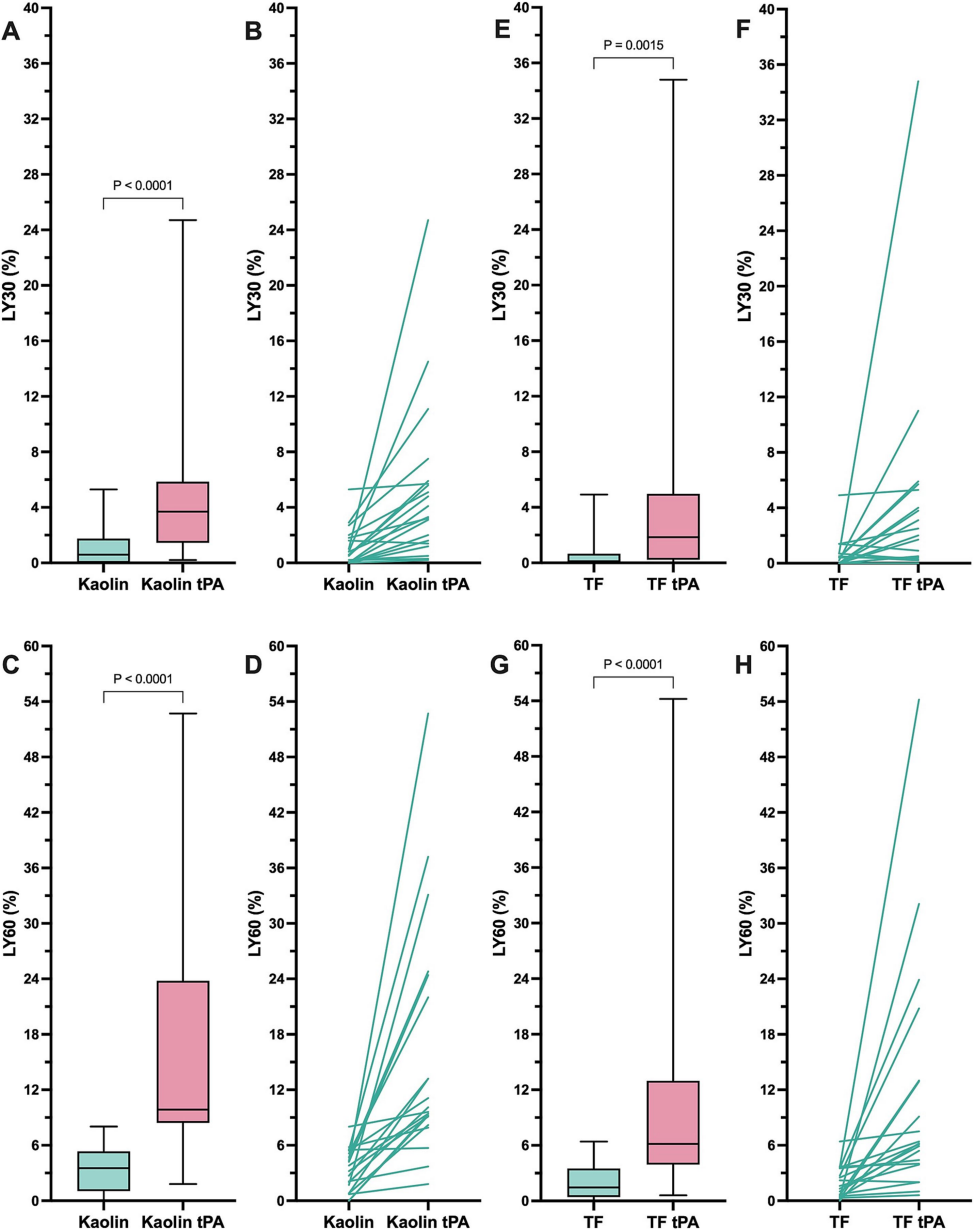
Kaolin and tissue factor activated thromboelastography assays with and without additional tissue plasminogen activator indicate that dogs with sepsis are hypofibrinolytic and display fibrinolysis resistance. **(A,B)** Kaolin LY30 +/− tPA. **(C,D)** Kaolin LY60 +/− tPA. **(E,F)** TF 1:3,600 LY30 +/− tPA. **(G,H)** TF 1:3,600 LY60 +/− tPA. Comparisons were performed using Wilcoxon signed rank tests. **(A,C,E,G)** Central horizontal line represents the median, the boxes represent the interquartile range, and the whiskers represent the minimum and maximum values. **(B,D,F,H)** Paired values are connected by lines to indicate the direction of change in the lysis values without and with additional tPA as indicated.

**Figure 2:**
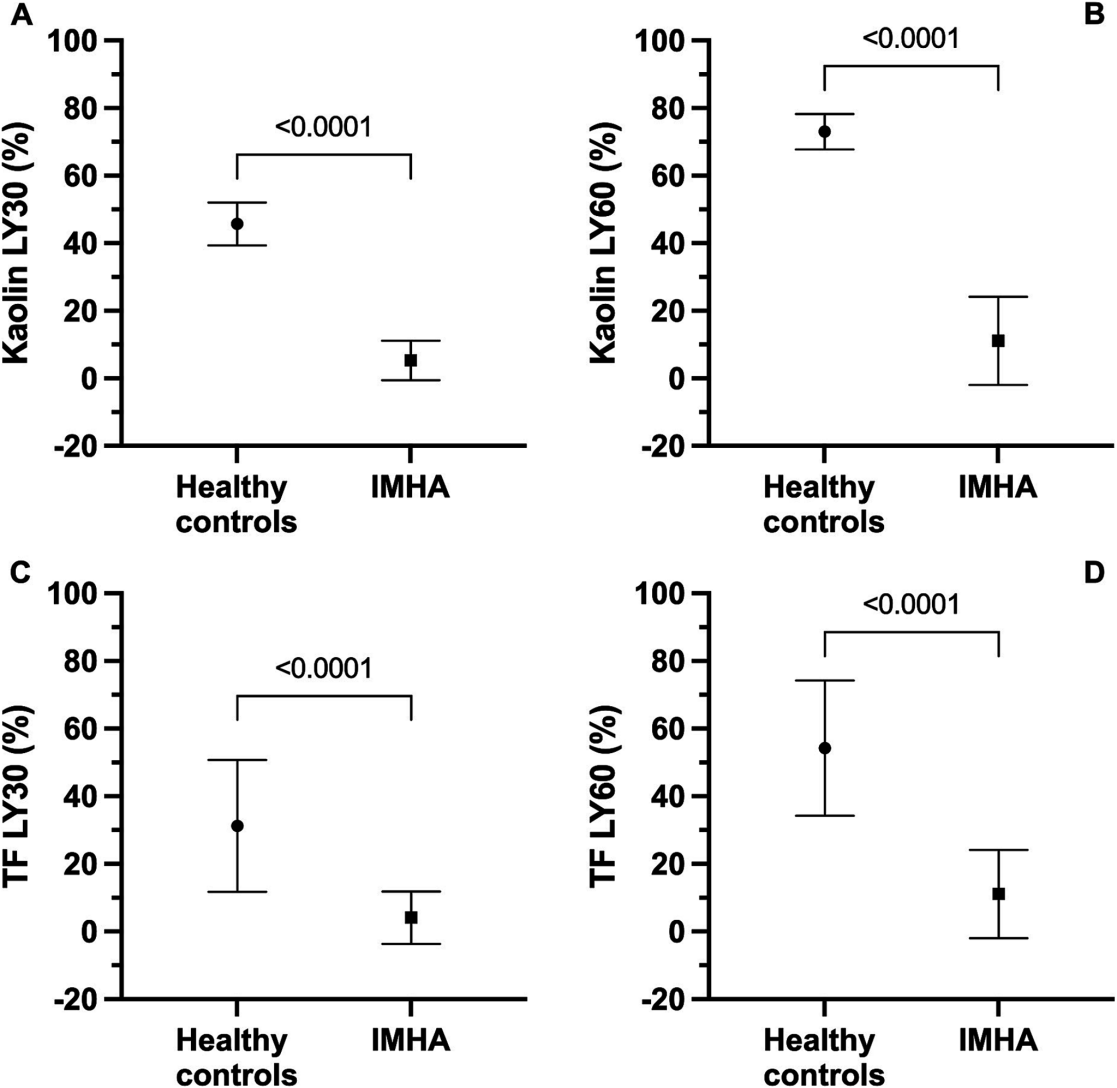
Kaolin and tissue factor (TF) activated thromboelastography assays with and without additional tissue plasminogen activator (tPA) indicate that dogs with sepsis are hypofibrinolytic and display fibrinolysis resistance. Note, healthy control data for the kaolin assay are derived from Spodsberg et al. (22), and healthy control data for the TF assay are derived from Fletcher et al. (49). All panels compare values in healthy controls with those in dogs with IMHA. **(A)** Kaolin LY30. **(B)** Kaolin LY60. **(C)** TF 1:3,600 LY30. **(D)** TF 1:3,600. For all panels, symbols represent the mean value, whiskers represent the standard deviation. Comparisons were performed using unpaired *t*-tests with Welch’s correction.

**Figure 3:**
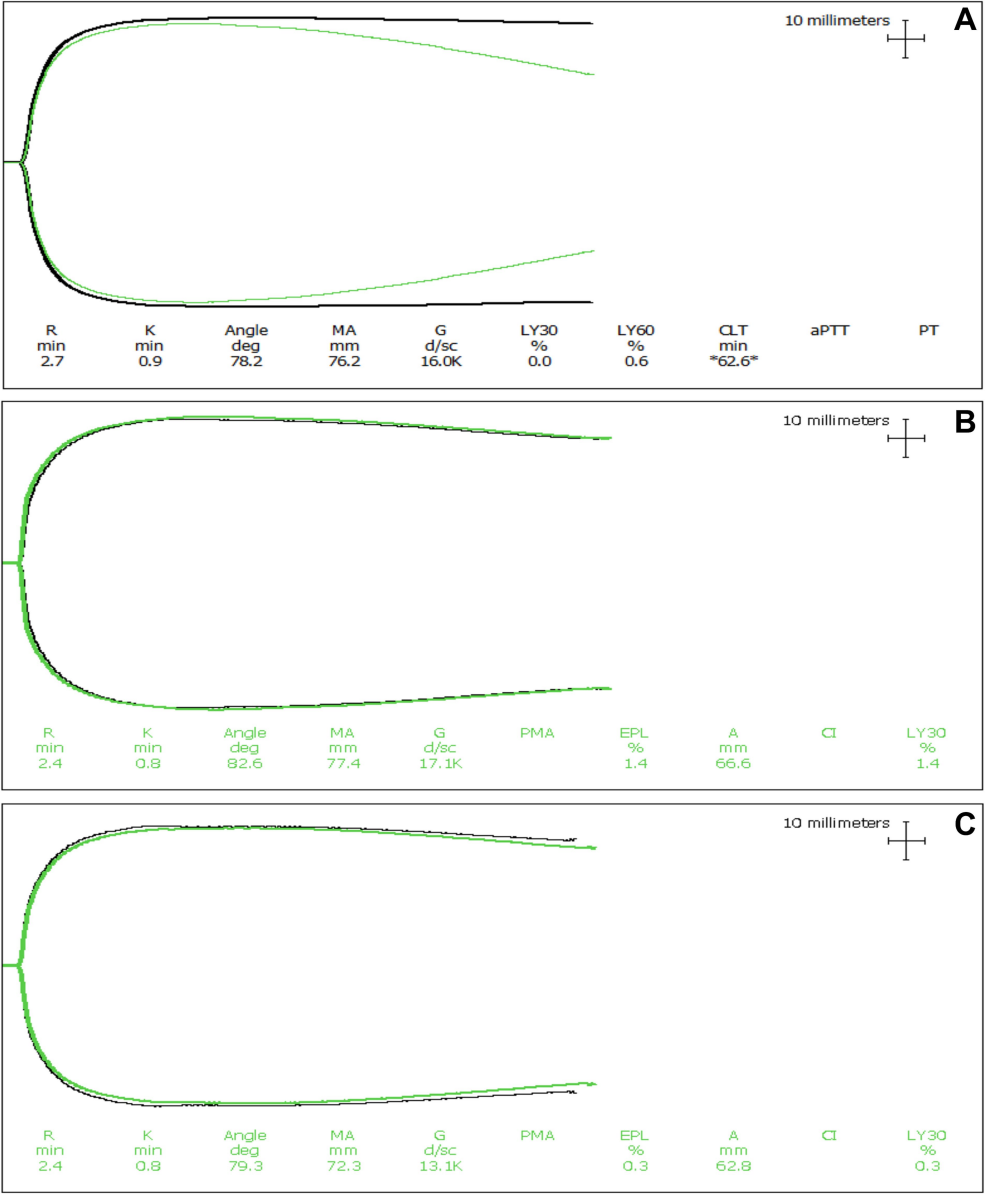
Representative thromboelastography (TEG) tracings of a dog with expected tPA-induced fibrinolysis. **(A)** Tissue factor-activated TEG tracings from a dog with IMHA where hypofibrinolysis was not apparent. The black line is the tracing with only the activator, while the green line is the tracing with both the activator and the addition of tPA. In these tracings, the addition of tPA caused clot lysis to occur relative to the paired tracing without the addition of tPA. Although the degree of fibrinolysis is diminished compared to healthy dogs, these tracings indicate that this dog did not have fibrinolytic shutdown. **(B,C)** Kaolin activated and tissue factor activated TEG tracings from dogs with IMHA displaying fibrinolysis resistance (also termed fibrinolytic shutdown). The black lines are the tracings with only the activator, while the green lines are the tracings with both the activator and the addition of tPA. In dogs with hypofibrinolysis (also termed fibrinolysis resistance or fibrinolytic shutdown) the addition of tPA does not cause clot lysis to occur.

In the kaolin- and TF-activated assays without tPA, 18/20 dogs with IMHA had hypercoagulable tracings relative to local reference intervals or published normal values (Supplementary Figures S1, S2) (49). In paired kaolin- and TF-activated thromboelastography profiles with and without additional tPA, there were significant increases in the clot lysis values at 30 and 60 min (LY30 and LY60) with the addition of tPA (Figure 1). There were also significant reductions in the maximum amplitude of the tracings with the addition of tPA (Tables 4, 5). Compared to the lowest values established for healthy controls, 19/20 dogs with IMHA demonstrated hypofibrinolysis and correspondingly, the tPA induced LY30 and LY60 values in dogs with IMHA were significantly lower than those reported for healthy dogs (Figure 2) (22, 49). When fibrinolysis resistance was defined by comparisons between paired tracings with and without additional tPA, 16/20 dogs had <5% difference between the LY30 values and 10/20 dogs had <1% difference (Figure 3).

**Table 4:** Summary data for the kaolin-activated thromboelastography with and with added tPA.

TEG variable	Kaolin	Kaolin + tPA	Adjusted *p*
Split point (SP, min)	2.0 (1.5–2.6)	1.9 (1.5–2.3)	0.1705
Reaction time (R, min)	2.2 (1.8–2.8)	2.1 (1.7–2.7)	**0.0002**
Clot formation time (K, min)	0.8 (0.8–0.9)	0.8 (0.8–0.8)	0.3371
Clot formation angle (α, °)	80.8 (77.6–82.1)	82.0 (78.6–83.2)	0.3371
Maximum amplitude (MA, mm)	76.4 (69.1–85.5)	74.0 (63.9–82.9)	**0.0315**
Maximum clot firmness (G, kdyne/s)	16.21 (11.19–29.56)	14.25 (8.87–24.17)	**0.0315**
Percent lysis at 30 min (LY30, %)	0.6 (0.0–1.8)	3.7 (1.5–5.9)	**<0.0001**
Percent lysis at 60 min (LY60, %)	3.5 (1.0–5.3)	9.9 (8.4–23.8)	**<0.0001**

The paired TEG variables with and without tPA were compared using Wilcoxon signed-rank tests corrected for multiple comparisons using the Holm–Šídák method. Values in bold type are statistically significant after adjustment for multiple comparisons.

**Table 5:** Summary data for the tissue factor-activated thromboelastography with and without added tPA.

TEG variable	Tissue factor	Tissue factor + tPA	Adjusted *p*
Split point (SP, min)	1.5 (1.2–1.9)	1.5 (1.2–1.9)	0.2774
Reaction time (R, min)	1.8 (1.5–2.2)	1.8 (1.4–2.2)	0.9375
Clot formation time (K, min)	0.8 (0.8–1.0)	0.8 (0.8–1.0)	0.9375
Clot formation angle (α, °)	80.4 (77.2–81.6)	79.8 (76.2–81.6)	0.4235
Maximum amplitude (MA, mm)	76.7 (70.8–82.4)	71.9 (64.8–78.3)	**<0.0001**
Maximum clot firmness (G, kdyne/s)	16.47 (12.17–23.36)	12.76 (9.28–18.05)	**<0.0001**
Percent lysis at 30 min (LY30, %)	0.1 (0.0–0.7)	1.9 (0.2–5.0)	**0.0076**
Percent lysis at 60 min (LY60, %)	1.5 (0.4–3.5)	6.2 (3.9–13.0)	**<0.0001**

The paired TEG variables with and without tPA were compared using Wilcoxon signed-rank tests corrected for multiple comparisons using the Holm–Šídák method. Values in bold type are statistically significant after adjustment for multiple comparisons.

### Coagulation testing

**Figure 4:**
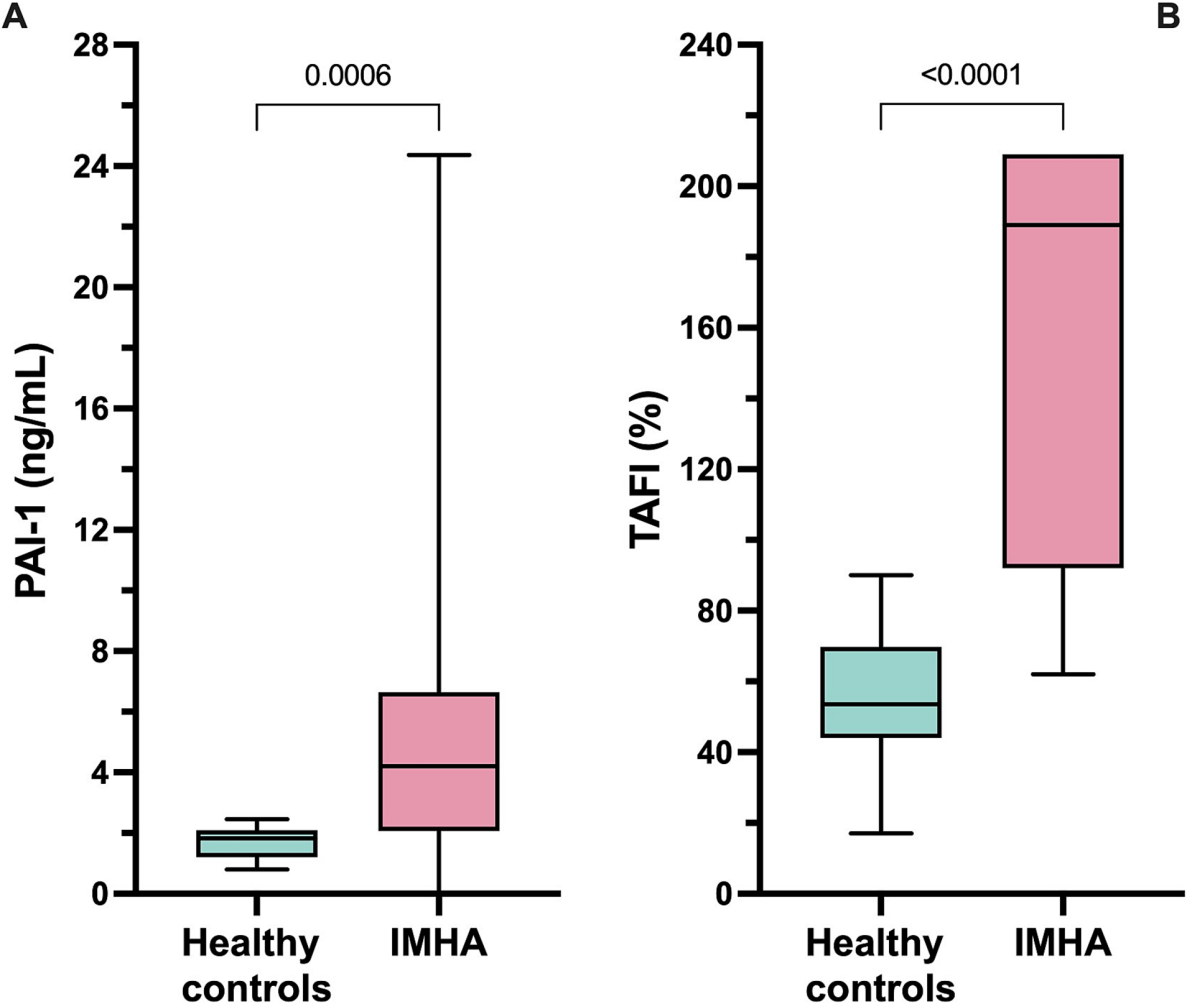
Box and whisker plots of **(A)** plasma concentrations of active plasminogen activator inhibitor-1 (PAI-1) and **(B)** plasma activity of thrombin-activatable fibrinolysis inhibitor (TAFI). Dogs with IMHA had significantly higher PAI-1 concentrations and TAFI activities than healthy controls. Comparisons were performed using the Mann–Whitney *U* test. The central horizontal line represents the median, the boxes represent the interquartile range, and the whiskers represent the minimum and maximum values.

**Figure 5:**
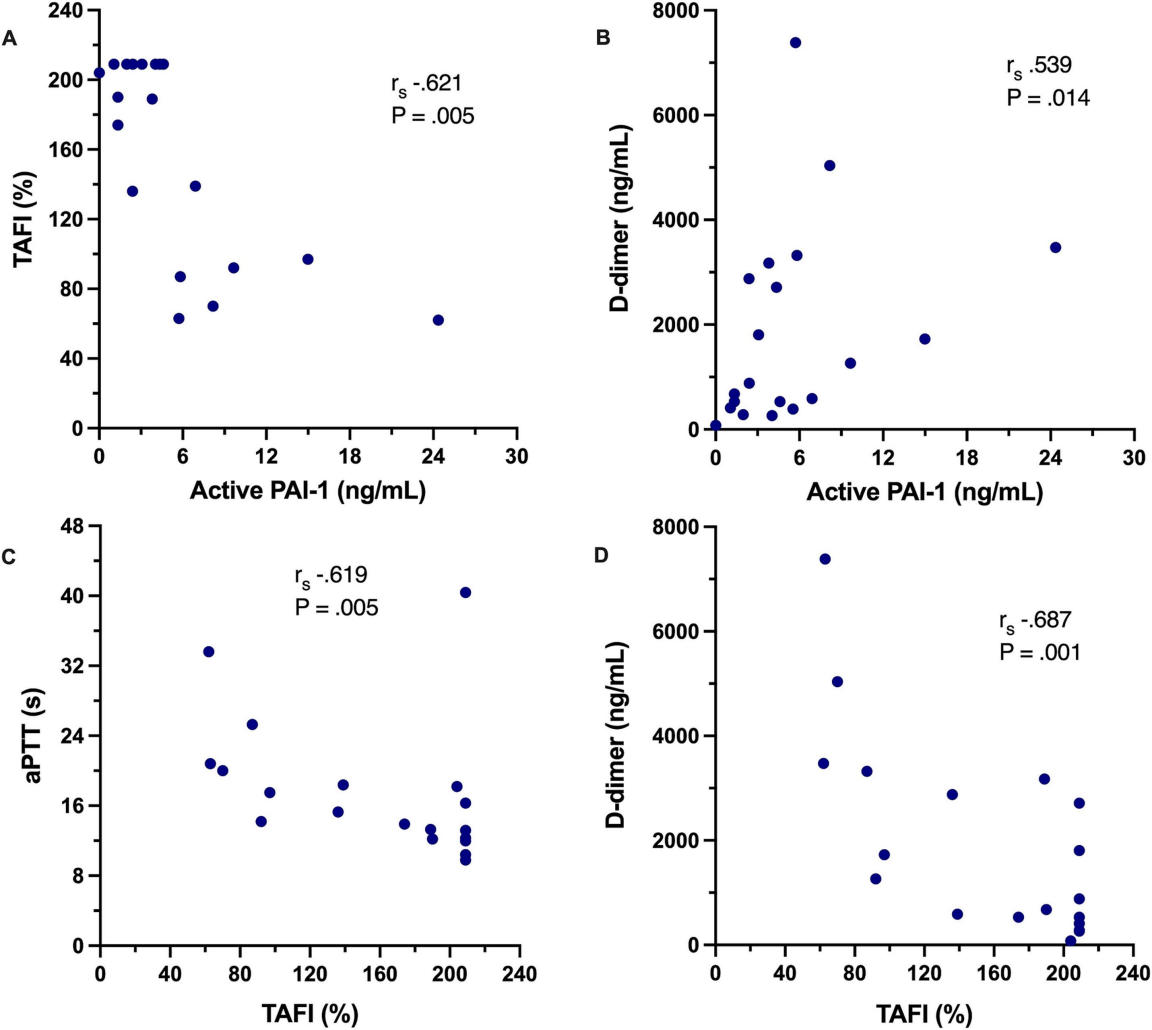
Scatterplots depicting the correlations between select coagulation variables in dogs with IMHA. **(A)** Plasma concentration of active plasminogen activator inhibitor-1 (PAI-1) against plasma activity of thrombin-activatable fibrinolysis inhibitor (TAFI). **(B)** Plasma concentration of active plasminogen activator inhibitor-1 (PAI-1) against plasma D-dimer concentration. **(C)** Plasma activity of thrombin-activatable fibrinolysis inhibitor (TAFI) against activated partial thromboplastin time (aPTT). **(D)** Plasma activity of thrombin-activatable fibrinolysis inhibitor (TAFI) against plasma D-dimer concentration. On each panel, the Spearman’s rank correlation coefficient (*r*_s_) and associated *p*-value are displayed.

Coagulation test results are summarized in Table 6. Dogs with IMHA had variably prolonged clotting times (aPTT, *n* = 8; PT, *n* = 3), hyperfibrinogenemia (*n* = 16) and increased D-dimer concentration (*n* = 11). Compared to healthy controls, dogs with IMHA had higher active PAI-1 concentrations [1.8 ng/mL (1.2–2.1) vs. 4.2 ng/mL (2.1–6.6), *p* < 0.001]. Dogs with IMHA also had higher TAFI activities than healthy controls [54% (44–70) vs. 189% (92–209), *p* < 0.0001, Figure 4]. To assess the associations of PAI-1 concentration and TAFI activity with the coagulation disturbances observed in dogs with IMHA, concentrations of PAI-1 and TAFI activities were compared with the fibrinolysis parameters derived from TEG (ΔCL30, ΔLY30, ΔCL60, ΔLY60) and with plasma coagulation assay values. Concentrations of PAI-1 were moderately negatively correlated with TAFI activity (*r*_s_ − 0.621, *p* = 0.005) and mildly correlated with D-dimer concentrations (*r*_s_ 0.539, *p* = 0.014); TAFI activity was moderately negatively correlated with D-dimer concentration (*r*_s_ − 0.687, *p* = 0.001), and aPTT (*r*_s_ − 0.619, *p* = 0.005, Figure 5). No correlations between the TEG delta fibrinolysis parameters and either PAI-1 or TAFI were identified.

**Table 6:** Summary of plasma coagulation test results.

Variable	Patient values	Reference interval
aPTT (s)	14.8 (12.5–19.6)	8.5–15.5
PT (s)	13.7 (12.8–14.9)	11.0–15.5
Fib (mg/dL)	1,099 ± 371	150–490
AT (%)	99 ± 28	65–145
D-dimer (ng/mL)	1,072 (441–3,101)	0–575

aPTT, activated partial thromboplastin time; AT, antithrombin activity; Fib, fibrinogen; PT, prothrombin time.

### NETosis biomarkers

Median plasma cfDNA concentrations of 1960 ng/mL (1,698–2,237) were significantly increased in dogs with IMHA compared with previously reported values of healthy controls 371 ng/mL (306–441) (*p* < 0.0001) (54). Median nucleosome concentrations 4.5 (2.1–13.7) were also increased relative to the pooled normal control value. Concentrations of these two biomarkers were not significantly correlated with each other and no significant correlations were observed between these biomarkers and the TEG delta fibrinolysis parameters or with active PAI-1 concentration or TAFI activity.

## Discussion

The high incidence and severe consequences of thrombotic complications in dogs with IMHA have been recognized for more than 30 years, yet treatment regimens for effective thromboprophylaxis remain undefined (55, 56). Enzymatic hypercoagulability, hyperfibrinogenemia, and intravascular TF expression have been identified in dogs with IMHA as contributory factors to development of pathologic thrombosis. The present study focused on hypofibrinolysis and NETosis as two additional prothrombotic phenomena and potential drug targets for this patient population. It was hypothesized that NETosis and hypofibrinolysis both occur in dogs with IMHA and are mechanistically linked. To investigate these hypotheses, 20 dogs with primary non-associative IMHA were enrolled, concentrations of two NETosis biomarkers measured, paired conventional and tPA-modified TEG assays performed, and activities of two plasma fibrinolysis inhibitors quantitated.

Abnormal plasma coagulation assay results were frequent in the dogs with IMHA in this study, with hyperfibrinogenemia present in 80% dogs, D-dimers increased in 55% dogs and at least 1 prolonged clotting time in 40%. These findings are consistent with other studies of dogs with IMHA (4, 5, 57, 58). Fibrinogen is a positive acute phase protein (59–61). The hyperfibrinogenemia most likely reflects the pro-inflammatory nature of IMHA in dogs that has been documented through immunophenotyping and studies of cytokine concentrations (62–64). Other acute phase proteins such as C-reactive protein were not measured in this study (23), but most dogs enrolled had a leukocytosis, primarily due to neutrophilia with left shift. The leukocyte changes in IMHA have been assessed as potential prognostic markers (65). The increased D-dimer concentrations indicate that thrombin activation and subsequent breakdown of cross-linked fibrin was occurring in the dogs in the present study (66). High D-dimer concentrations might be indicative of pathologic thrombosis (67) and disseminated intravascular coagulation (51, 68) and are associated with disease severity and poor prognosis in dogs with IMHA (69). Prolongation of the aPTT is also a frequent occurrence in critically ill humans (70) and dogs (71) and could result from multiple decreased factor activities, an increase in inhibitors such as antiphospholipid antibodies (72) or the effects of inflammation on clot-based *in vitro* assays (73). None of the coagulation assay abnormalities observed are specific to IMHA. Such changes might be more frequent in dogs with thrombosis (17), but they are not always diagnostic (74).

To further investigate the hemostatic imbalance in dogs with IMHA, paired TEG assays with and without additional tPA were used to increase the sensitivity of the TEG platform for detection of fibrinolysis resistance. With both kaolin and TF activation, dogs with IMHA had rapid clot formation (short R- and K-times and steep alpha angles) and generated firm clots (high MA and G values). These findings are typical of dogs with IMHA (19–21), and are likely the consequence of hyperfibrinogenemia, platelet activation, and the effect of anemia on the proportions of plasma and cells within the fixed reaction volume (75, 76). Hypofibrinolysis, as measured by tPA-TEG, was very common in dogs with IMHA. When the definition of hypofibrinolysis involved comparisons to pre-existing healthy control data (22), fibrinolysis resistance was present in 19/20 dogs. To avoid reliance on historical control data, fibrinolysis resistance was considered present when the addition of tPA induced minimal fibrinolysis. If the threshold was set at <5% lysis, then 80% of dogs were tPA resistant. When the threshold was set at 1% lysis, 50% of dogs were deemed tPA resistant. In the paired TEG assays, the addition of tPA to the reaction significantly increased LY30 and LY60 values but also reduced MA and G. The tPA was added to the reaction mixture before the assay was initiated and thus was present throughout the processes of clot formation and lysis. As such, the tPA could have generated plasmin from plasminogen as fibrin strands were forming, thereby initiating lysis and reducing the total amount of fibrin present at the time of MA. The median MA values of the tracings with tPA present were within ~94% of the values without tPA, suggesting that even with the addition of tPA, the balance remained in favor of thrombin generation promoting fibrin clot formation. The presence of tPA might also have altered the nature of the fibrin clots formed within the assay cup, by reducing their density and complexity through alterations in fibrin strand configuration (77). In turn, that could have impacted interactions with enmeshed platelets and erythrocytes and reduced clot strength (78–80). Observations of fibrin clot formation in the presence of varying amount of tPA suggest that when present throughout clot formation, tPA alters the structure of the resulting fibrin meshwork (81). Thrombin generated within the assay cup converts the available fibrinogen into a branched fibrin polymer network (82). The functional properties of the fibrin meshwork and its susceptibility to fibrinolysis are the result of variations in the ultrastructure of the mesh. Fibrin fibers vary in length, diameter and degree of branching, which affect its mechanical properties (83). This variation results from the concentrations of fibrinogen and thrombin, the presence of extracellular DNA (84), and reactive oxygen species (85). In general, plasma from humans with thrombotic disorders form plasma clots *in vitro* that are denser, less permeable and more rigid. These clots are more resistant to fibrinolysis than those formed from the plasma of healthy individuals (86). Alterations in fibrin clot ultrastructure determine susceptibility to fibrinolysis and can influence disease outcomes including thrombotic risk (87–89), and such abnormalities have been documented in immune-mediated diseases in humans including antiphospholipid syndrome (90), rheumatoid arthritis (91), and systemic lupus erythematosus (92). The nature of the clots that form under different conditions has implications for the efficacy of endogenous fibrinolysis, and the therapeutic potential of exogenous thrombolytic drugs (93). Indeed, use of scanning electron microscopy might be of value to provide morphological insights in future studies of thrombosis in dogs with IMHA (94).

Consistent with the TEG findings, the increased concentrations of active PAI-1 suggest that dogs with IMHA have a fibrinolytic imbalance that could impair clot breakdown and potentiate the thrombotic risk. The increased active PAI-1 concentrations observed extend the findings of a previous report that documented increased PAI-1 mRNA in dogs with IMHA (95). PAI-1 is the main physiological inhibitor of tPA and urokinase and is produced by endothelial cells, platelets and monocytes (96). Few studies of PAI-1 have been reported in dogs to date but a previous study using a different assay, observed increased PAI-1 activity in hyperlipidemic dogs with hyperadrenocorticism and diabetes mellitus (97). Increased total plasma PAI-1 concentrations have also been observed in dogs with sepsis (98). In the present study, active PAI-1 concentrations were correlated with D-dimer concentrations, but not with the ΔCL or ΔLY values derived from comparisons of TEG tracings with and without tPA. These parameters were used for correlation assessments to look for potential mechanistic insights into the fibrinolysis resistance identified. It was judged that those parameters best described the effect of tPA on clot formation in the TEG assay.

The activity of the fibrinolysis inhibitor TAFI was increased in dogs with IMHA in this study. Increased TAFI concentrations are associated with increased risk of venous thrombosis and stroke in humans (99–101) and have been reported in dogs with babesiosis (102) and sepsis (52, 103). Released from the liver in an inactive form, active TAFI is typically generated through zymogen cleavage by the thrombin-thrombomodulin complex. Active TAFI then removes C-terminal lysine residues from fibrin, limiting formation of the fibrin-plasmin-tPA ternary complex. This limits plasmin generation and thereby suppresses fibrinolysis (104). The increased TAFI activity in dogs with IMHA might have resulted from increased hepatic synthesis (105) potentially in combination with reduced endothelial cell thrombomodulin expression (106), with a resultant imbalance between production and consumption. Platelets and megakaryocytes also store and release TAFI, although the relative contribution of platelets to plasma TAFI concentrations is small (107). As with PAI-1, none of the TEG ΔCL or ΔLY values correlated with TAFI activities. This could imply that plasma PAI-1 and TAFI are not the major factors inducing fibrinolysis resistance measured by TEG or that the tPA-TEG assay is not a sensitive quantitative measure of PAI-1 or TAFI activities, perhaps because tPA concentration in the TEG assay is supraphysiologic.

Consistent with a previous report (35) increased plasma cfDNA and nucleosome concentrations in dogs with IMHA were observed. These markers were used as surrogates for NETosis but recognize that there are other potential sources of both, including apoptosis and necrosis. No ideal biomarker exists for NETosis currently, and efforts to clearly determine the extent of this phenomenon are hampered by the lack of a quantitative assay for citrullinated histones, purported to be highly specific for NETosis detection in humans (108). Future studies might incorporate immunofluorescence or confocal microscopy for visual confirmation of NET formation as has been described for dogs with sepsis (109, 110), and cats with cardiomyopathy (111).

It was hypothesized that the hypofibrinolysis in dogs with IMHA was related to the extent of NETosis. It was therefore surprising to find no significant association between the TEG lysis parameters and concentrations of cfDNA or nucleosomes. Although cfDNA and nucleosomes might be imperfect NETosis biomarkers, the presence of high concentrations of these molecules might be expected to alter the fibrinolytic potential of dogs with IMHA irrespective of their cellular origin. The most likely explanation for the lack of association between these biomarkers and the TEG parameters is that the TEG assay is insensitive to the presence of these molecules. This suggests that future investigations of the association between NETosis markers and fibrinolysis in dogs might need to employ other screening tests such as the overall hemostatic potential assay (112, 113).

It might be that the nature of shear force generation within the TEG assay limits its sensitivity to fibrinolysis disorders. Thromboelastography is termed a viscoelastic test of coagulation because both viscosity and elasticity contribute to clot amplitude (114, 115). The TEG 5000 assay platform employed in the present study used a standard 4°45′ oscillation angle with a 10s cycle time (0.1 Hz). This configuration generates very low strain rates reported to be 0.5 s^−1^ (116) or as low as 0.03 s^−1^ (117). The extent and rate of cup oscillation was consistent during each experiment and throughout the study. However, the strain amplitude applied by the cup/pin assembly on the forming clot varies, decreasing as the clot forms (115). It is reported that clots demonstrate linear viscoelastic behavior only when the shear strain is <2% (118). In typical TEG measurements configured as above where the MA is ~50 mm, the shear strain varies from 16% at the start of the tracing to 8% at MA (118). The shear strain at the higher MA values in the present study would have been lower than 8%, but likely still >2%. The oscillating motion of the TEG cup/pin can substantially delay clot formation and modify clot structure, ultimately leading to generation of weaker clots relative to those formed under static conditions (118, 119). These physical effects might help explain the apparent insensitivity of the TEG assays to detect fibrinolysis in these dogs and the lack of association between TEG assay variables and fibrinolysis inhibitor activities. The mapping of the clot lysis phase of the TEG tracing is also dependent upon the interaction of the clot with the rotating cup. If a robust clot forms and subsequent wall slippage occurs (120), then an apparent insensitivity to clot lysis could result, and may have contributed to the observations in the present study.

Another possible explanation for the limited fibrinolysis seen in the present study is the effects of platelets. Others have demonstrated using thromboelastography, that resistance of blood clots to t-PA-induced fibrinolysis is related to platelet number and function (121). It was demonstrated that platelets inhibited fibrinolysis through activation of TAFI and clot retraction. The nature of the fibrin meshwork also influences the degree to which platelets can contract clots (122, 123). In the TEG cup, platelet-mediated clot retraction may reduce interactions between the cup and the pin thereby limiting the ability of the system to identify breakdown of the fibrin strands.

The present study has limitations. Based on sample size calculations only a small number of dogs with IMHA were enrolled. These dogs were clinically homogeneous in that they were typically newly diagnosed, non-associative IMHA cases, often with severe disease as indicated by the median CHAOS score. These factors could limit the generalizability of these results. The study was conducted over an extended period, in large part due to the COVID-19 pandemic that began shortly after the study was initiated and severely restricted case recruitment. The extended duration of the study would not have impacted the TEG assay data, because these tests were performed as paired analyses shortly after sample collection. However, the samples that were collected and stored pending batch analyses were frozen for extended and inconsistent periods of time. It is possible that some degradation of analytes occurred during prolonged storage and influenced the measured concentrations and activities impacting attempts to identify relationships between variables. Fibrinolytic factors are very stable when stored at −80°C for periods >5 years (124). High cfDNA and nucleosome concentrations were identified, however, and increased PAI-1 and TAFI activities despite the prolonged storage, arguing against substantial degradation of these biomarkers during storage. Many of the dogs in the present study had hyperbilirubinemia, including six dogs with values >2.0 mg/dL, and six dogs had severe hemolysis based on hemolysis index. These abnormalities are typical of dogs with severe IMHA and comparable to previously published studies. The TEG assays and the clot endpoint coagulation assays would not have been affected by either of these substances. The PAI-1 assay might have been influenced by the hemoglobin or bilirubin present in some samples, however, because that assay uses a chromogenic substrate with light absorbance measured at 450 nm. Hemoglobin absorbs light most strongly at 550 nm and 420 nm (125). While this does not directly correspond with the wavelength used for the PAI-1 assay, spectral overlap might increase the apparent PAI-1 concentration in the most severely hemolyzed samples. Bilirubin absorbs light most strongly around 450 nm (126), and hence would overlap with the PAI-1 assay wavelength. As such, PAI-1 concentrations might have been overestimated in a small number of dogs in this study. The TAFI assay also uses a chromogenic substrate, but absorbance is detected at a shorter wavelength (405 nm). Moreover, TAFI activity is derived from a curve plotted using quantitative rate of change in absorbance values such that the presence of hemoglobin or bilirubin in the samples has less influence on calculated values. The assay manufacturer also states that hemolysis does not interfere with the performance of the assay.

Data from the present study support the hypothesis that hypofibrinolysis, characterized by resistance to tPA activated whole blood clot lysis, is a common feature of IMHA in dogs. Dogs with IMHA had significantly higher plasma active PAI-1 concentration and TAFI activities that could have contributed to the observed hypofibrinolysis. Moreover, identification of increased active PAI-1 concentrations in dogs with IMHA further supports prior transcriptomic data of increased PAI-1 expression in these patients and suggests novel therapeutic opportunities using PAI-1 inhibitors (127–129). The combined hypercoagulability and hypofibrinolysis common in dogs in this study supports recent recommendations to provide thromboprophylaxis to all dogs with IMHA (55, 130). The observed increases in NETosis biomarkers also suggest that formation of NETs contributes to the pathophysiology of IMHA in dogs and that NETs might represent novel therapeutic targets for interventions such as DNAse and non-anticoagulant heparin (36).

## Data Availability

The raw data supporting the conclusions of this article will be made available by the authors, without undue reservation.

## Supplementary material

SUPPLEMENTARY DATA SHEET S1 Detailed protocols for the conduct of the tissue plasminogen activator (tPA) augmented thromboelastography (TEG) assays.

SUPPLEMENTARY FIGURE S1 Dotplots of the five principal values derived from the kaolin-activated thromboelastography tracings with no additional tissue plasminogen activator (tPA). **(A)** Reaction time (R time). **(B)** Clot formation time (K time). **(C)** Alpha angle (clot formation angle). **(D)** Maximum amplitude (MA). **(E)** Maximum clot firmness (G). Horizontal dotted lines represent the institutional reference intervals for this assay.

SUPPLEMENTARY FIGURE S2 Dotplots of the five principal values derived from the tissue factor (TF) activated thromboelastography tracings with no additional tissue plasminogen activator (tPA). The final in cup dilution of TF was 1:3,600. **(A)** Reaction time (R time). **(B)** Clot formation time (K time). **(C)** Alpha angle (clot formation angle). **(D)** Maximum amplitude (MA). **(E)** Maximum clot firmness (G). Horizontal dotted lines represent the minimum and maximum values from healthy control dogs derived from Fletcher et al. (49) for this assay configuration.

## Glossary

α: Clot-formation angle
AHDC: Animal Health Diagnostic Center
APPLE: Acute patient physiologic and laboratory evaluation
aPTT: Activated partial thromboplastin time
AT: Antithrombin
CBC: Complete blood count
cfDNA: Cell-free DNA
CHAOS: Canine hemolytic anemia objective score
CL30: Clot lysis at 30 min
CL60: Clot lysis at 60 min
G: Clot firmness
IMHA: Immune-mediated hemolytic anemia
K: Clot formation time
LY30: Percentage clot lysis at 30 min
LY60: Percentage clot lysis at 60 min
MA: Maximum amplitude
NETs: Neutrophil extracellular traps
PAI-1: Plasminogen activator inhibitor-1
PCV: Packed cell volume
PT: Prothrombin time
R: Reaction time
*r*_s_: Spearman’s rank correlation coefficient
TAFI: Thrombin activatable fibrinolysis inhibitor
TEG: Thromboelastography
TF: Tissue factor
tPA: Tissue plasminogen activator
