# Supplementary material for: Tissue plasminogen activator modified thromboelastography identifies fibrinolysis resistance in dogs with immune-mediated hemolytic anemia
Source: Front Vet Sci. 2025 Jun 5;12:1571683. doi: 10.3389/fvets.2025.1571683 (PMC12177219; doi:10.3389/fvets.2025.1571683)
Supplement: SUPPLEMENTARY DATA SHEET S1 — Detailed protocols for the conduct of the tissue plasminogen activator (tPA) augmented thromboelastography (TEG) assays. [file Data_Sheet_1.PDF]

IMHA-NETosis TEG protocol

Pre-study general procedures

Please check all channels using Level 1 and Level 2 QC samples per routine maintenance protocols.
The same experienced operator should perform all sample analysis.
All samples should be analyzed until LY60 values are obtained.
All available channels should be used simultaneously to expedite analysis.

TF reagent preparation

1. Prepare TF reagent immediately prior to use.
2. Reconstitute lyophilized Innovin using 4 mL sterile water for injection. Avoid foaming.
   After reconstitution, this **Innovin stock solution** can be stored at 4°C for 10 days
3. Thaw 2 mL aliquot of **TF dilution buffer** (2% BSA, 20mM HEPES, 150mM NaCl)
4. Add 40 μL Innovin stock solution to 360 μL of TF dilution buffer. Gently mix by inversion.
   This is solution **TF1**, keep on ice until required.
5. Add 50 μL of solution TF1 to 170 μL of TF dilution buffer. Gently mix by inversion.
   This is solution **TF2**, keep on ice until required.

tPA reagent preparation

1. Reconstitute 2 mg vial of lyophilized alteplase (Cathflo) with 2,000 μL sterile water
2. Final concentration 1 mg/mL (580,000 U/mL)
3. Freeze resulting **tPA stock solution** in 50 μL aliquots at –80°C for a maximum of 6 months
4. Thaw the tPA stock solution aliquot at room temperature
5. Add 40 μL of tPA stock solution to 424 μL sterile water, mix by pipetting. This is **tPA1**.
6. Add 18 μL of tPA1 to 482 μL sterile water, mix by pipetting. This is **tPA2**.

Method - Kaolin assay

Use a fresh, clean pipette tip for every stage

1. Allow one kaolin tube to equilibrate to room temperature (at least 5 minutes)
2. Allow one tPA aliquot to thaw at room temperature (at least 10 minutes)
3. Pre-warm two TEG cups (plain or heparinase depending on patient situation)
4. Load 20 μL of $CaCl_2$ into both
5. Into the first cup load 10 μL sterile water
6. Into the second cup load 10 μL **tPA2** (made as above)
7. Transfer 1000 μL of citrate whole blood into a kaolin vial
8. Invert the kaolin vial gently 5 times to mix the sample/kaolin
9. From the kaolin tube transfer one aliquot of **330** μL into each pre-warmed TEG cup
10. Gently mix $CaCl_2$ / tPA or water / kaolin-whole blood by 1x up/down pipette
11. For each channel, raise cup to pin, slide lever to test, click start in software to analyze

Method - Tissue factor (Innovin) 1:3,600 assay

Use a fresh, clean pipette tip for every stage

1. Allow one tPA aliquot to thaw at room temperature (at least 10 minutes)
2. Pre-warm two TEG cups (plain or heparinase depending on patient situation)
3. Load 20 μL of $CaCl_2$ into both
4. Into the first cup load 10 μL sterile water
5. Into the second cup load 10 μL **tPA2** (made as above)
6. Transfer 740 μL of citrate whole blood into an Eppendorf tube
7. Add 10 μL **TF2** to the citrate whole blood aliquot and mix by gentle inversion.
   This is **TF-whole blood**.
8. Load **330** μL of **TF-whole blood** to each TEG cup
9. Gently mix $CaCl_2$ / tPA or water / TF-whole blood by 1x up/down pipette
10. For each channel, raise cup to pin, slide lever to test, click start in software to analyze
