# Supplementary figures and images for: Tissue plasminogen activator modified thromboelastography identifies fibrinolysis resistance in dogs with immune-mediated hemolytic anemia
Source: Front Vet Sci. 2025 Jun 5;12:1571683. doi: 10.3389/fvets.2025.1571683 (PMC12177219; doi:10.3389/fvets.2025.1571683)

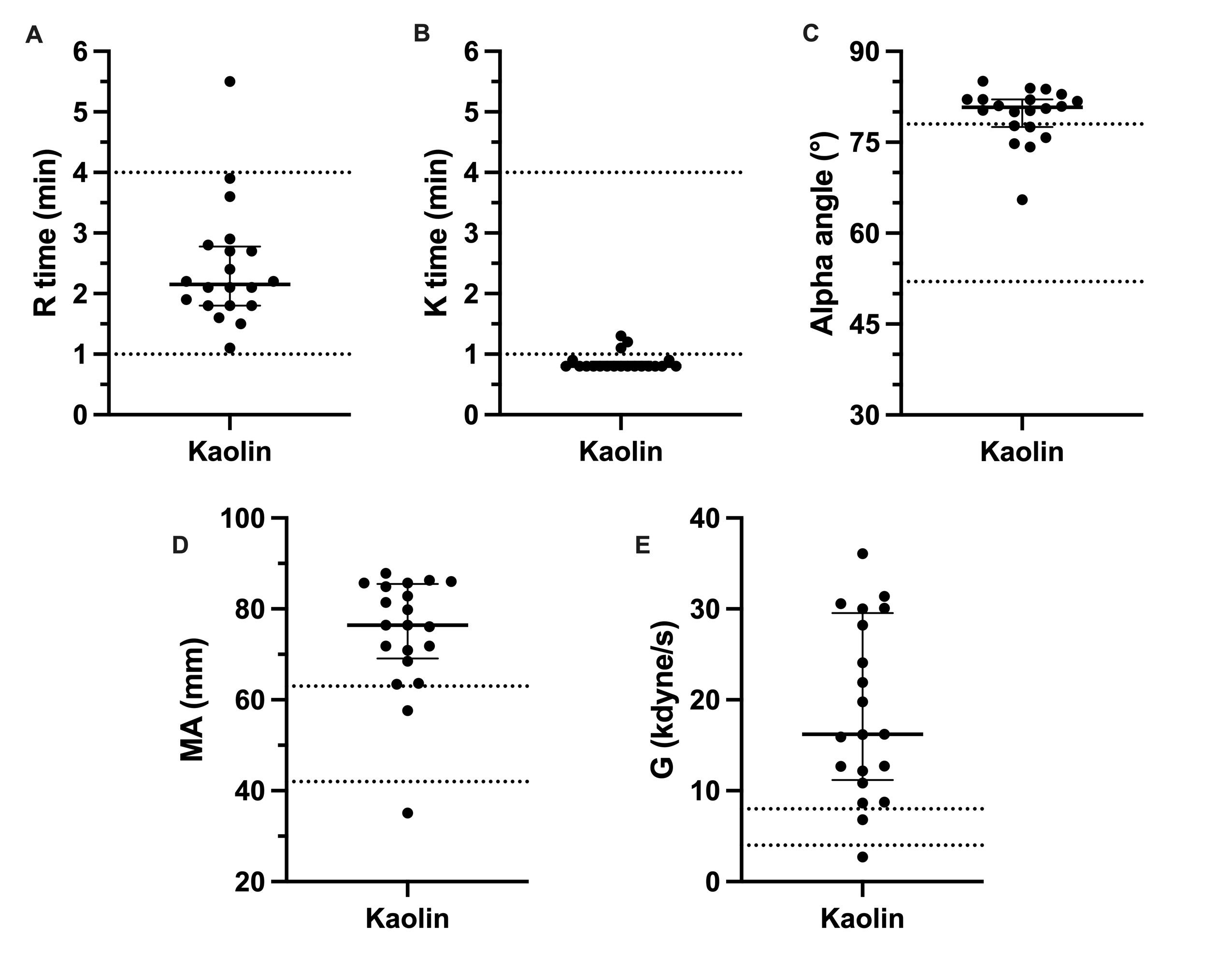

Supplement: SUPPLEMENTARY FIGURE S1 — Dotplots of the five principal values derived from the kaolin-activated thromboelastography tracings with no additional tissue plasminogen activator (tPA). (A) Reaction time (R time). (B) Clot formation time (K time). (C) Alpha angle (clot formation angle). (D) Maximum amplitude (MA). (E) Maximum clot firmness (G). Horizontal dotted lines represent the institutional reference intervals for this assay. [file Image_1.JPEG]

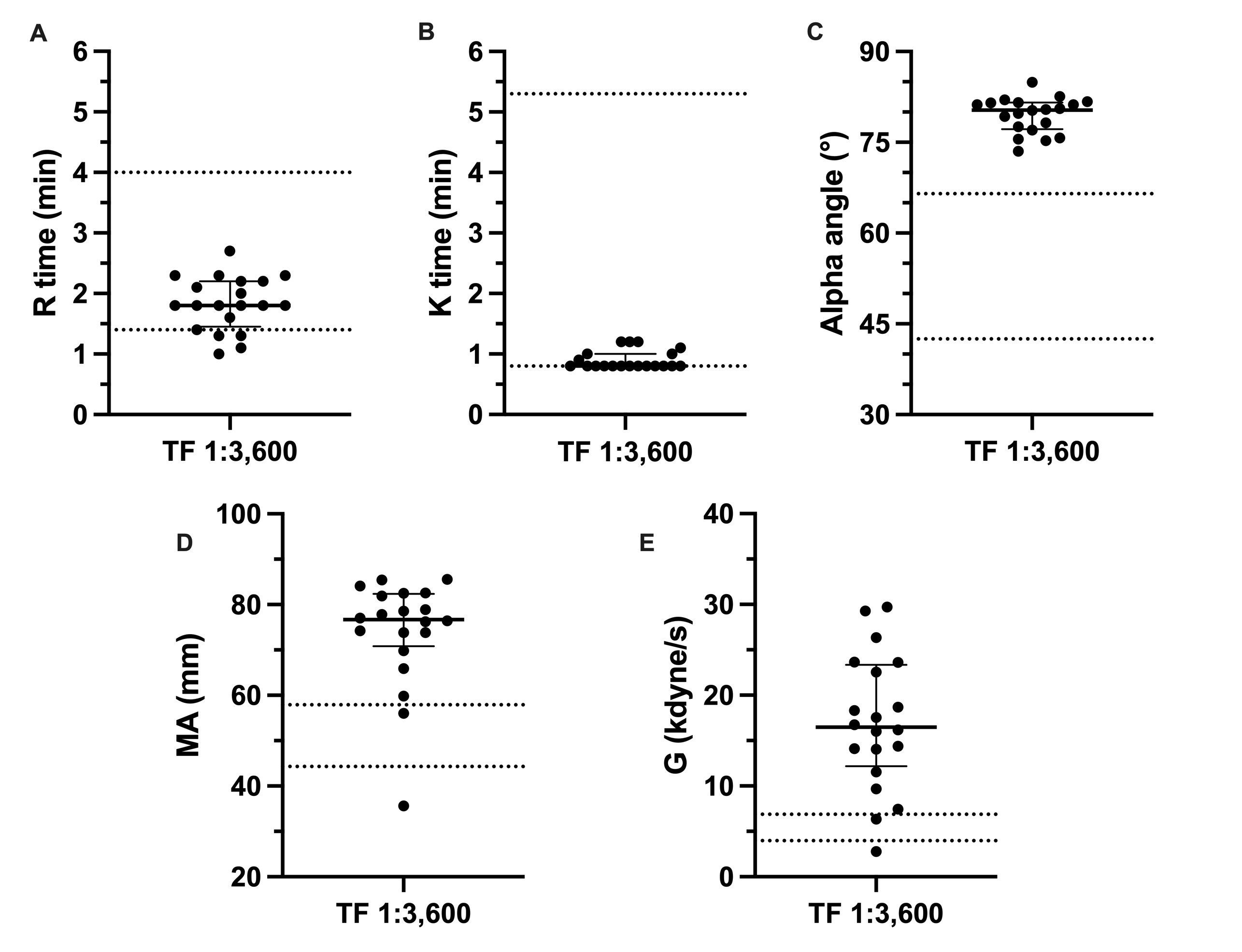

Supplement: SUPPLEMENTARY FIGURE S2 — Dotplots of the five principal values derived from the tissue factor (TF) activated thromboelastography tracings with no additional tissue plasminogen activator (tPA). The final in cup dilution of TF was 1:3,600. (A) Reaction time (R time). (B) Clot formation time (K time). (C) Alpha angle (clot formation angle). (D) Maximum amplitude (MA). (E) Maximum clot firmness (G). Horizontal dotted lines represent the minimum and maximum values from healthy control dogs derived from Fletcher et al. (49) for this assay configuration. [file Image_2.JPEG]
